# Correlation Kernels for Support Vector Machines Classification with Applications in Cancer Data

**DOI:** 10.1155/2012/205025

**Published:** 2012-08-07

**Authors:** Hao Jiang, Wai-Ki Ching

**Affiliations:** Advanced Modelling and Applied Computing Laboratory, Department of Mathematics, Run Run Shaw Building, The University of Hong Kong, Pokfulam Road, Hong Kong

## Abstract

High dimensional bioinformatics data sets provide an excellent and challenging research problem in machine learning area. In particular, DNA microarrays generated gene expression data are of high dimension with significant level of noise. Supervised kernel learning with an SVM classifier was successfully applied in biomedical diagnosis such as discriminating different kinds of tumor tissues. Correlation Kernel has been recently applied to classification problems with Support Vector Machines (SVMs). In this paper, we develop a novel and parsimonious positive semidefinite kernel. The proposed kernel is shown experimentally to have better performance when compared to the usual correlation kernel. In addition, we propose a new kernel based on the correlation matrix incorporating techniques dealing with indefinite kernel. The resulting kernel is shown to be positive semidefinite and it exhibits superior performance to the two kernels mentioned above. We then apply the proposed method to some cancer data in discriminating different tumor tissues, providing information for diagnosis of diseases. Numerical experiments indicate that our method outperforms the existing methods such as the decision tree method and KNN method.

## 1. Introduction

 In the current perspective, support vector machines (SVMs) demonstrate as benchmarks for various disciplines such as text categorization and time series prediction and they have gradually become popular tools for analyzing DNA microarray data [1]. SVMs were first used in gene function prediction problems and later they were also applied to cancer diagnosis based on tissue samples [2]. The effectiveness of SVMs depends on the choice of kernels. Recently correlation kernel with SVM has been applied successfully in classification. The correlation matrix gives the correlation coefficients among all the columns in a given matrix. To be precise, in a correlation matrix, the *ij*th entry measures the correlation between the *i*th column and *j*th column of a given matrix. The diagonal entries in the correlation matrix are all equal to one because they compute the correlation of all the columns with themselves. Furthermore, the correlation matrix is symmetric because the correlation between *i*th column and *j*th column is the same as the correlation between *j*th column and *i*th column in the matrix. There are several possible correlation coefficients, the most popular one is the Pearson correlation coefficient, see for instance [3]. In the case of a perfect positive linear correlation, the Pearson correlation coefficient will be 1. While −1 indicates a perfect negative anticorrelation. Usually the correlation coefficients lie in the interval (−1,1), indicating that the degree of linear dependence between the variables within a given matrix. An important property of the correlation matrix is that it is always positive semidefinite.

Correlation kernel with SVMs is a recent application in biological research. It can be effectively used for the classification of noisy Raman Spectra, see for instance [4, 5]. The construction of correlation kernel involves the use of distance metric which is problem specific but this is less common in kernel methods. Correlation kernel is self-normalizing and is also suitable for classification of Raman spectra with minimal pre-processing. The similarity metric defined in the kernel describes the similarities between two data instances. The positive semidefinite property of the usual correlation kernel is ensured if the correlation matrix itself is positive semidefinite.

The kernel matrices resulting from many practical applications are indefinite and therefore are not suitable for kernel learning. This problem has been addressed by various researchers, see for instance [6–9]. A popular and straightforward way is to transform the spectrum of the indefinite kernel in order to generate a positive semidefinite one. Representatives such as the *denoising* method which treats negative eigenvalues as ineffective [10]. The *flipping* method flips the sign of negative eigenvalues in kernel matrix [11]. The *diffusion* method transforms the eigenvalues to their exponential form [12] and the *shifting* method applies positive shift to the eigenvalues [13].

Taking into consideration that a correlation matrix is positive semidefinite, we can therefore construct a parsimonious kernel matrix such that the positive semidefiniteness is satisfied automatically. This novel kernel is so far until now the first application in classification problems. Apart from that, we also propose a kernel sharing similar expression with the usual correlation kernel. However, the *denoising* method was applied accordingly to construct a novel positive semidefinite kernel matrix. The reason why we choose the *denoising* method is that the technique has been successfully used in protein classification problem [14]. This suggests that it may have an important role in classification for other biological data sets.

The remainder of this paper is structured as follows. In Section 2, we introduce the construction of usual correlation kernel. We proposes the parsimonious positive semidefinite kernel as well as the novel kernel after *denoising* on a kernel having similar property with the usual correlation kernel. Theoretical proof on the positive semidefinite property of parsimonious kernel was provided. We also give explanations for particular property of related kernels. In Section 3, publicly available data sets are utilized to check the performance of the proposed method and compare to some state-of-the-art methods such as the KNN method and the decision tree method. A discussion on the results obtained is given in Section 4. Finally concluding remarks are given in Section 5.

## 2. The Proposed Parsimonious Positive Semi****definite Kernel Method

In this section, we first introduce the usual correlation kernel. Based on the positive semidefinite property of the usual correlation kernel, we then propose a parsimonious positive semidefinite kernel. Apart from that, our novel kernel, namely, DCB (denoised correlation based) kernel will be presented.

### 2.1. The Usual Correlation Kernel

In this section, we assume that there are *n* data instances in the data set. The number of features used to describe a data instance is *p*. Then the data matrix can be expressed as a *p* × *n* matrix which we denote as follows:

(1) X=[X1,X2,…,Xn],  Xi=[x1i,x2i,…,xpi]T,              i=1,2,…,n.

It is straightforward to obtain the correlation matrix of *X*. Here we suppose the correlation matrix is corr(*X*). Then we have

(2) $$\begin{array}{c} \text{corr}\left(X\right)=\left[\begin{array}{cccc} \text{corr}\left(X_{1},X_{1}\right) & \text{corr}\left(X_{1},X_{2}\right) & \cdots & \text{corr}\left(X_{1},X_{n}\right)\\ \text{corr}\left(X_{2},X_{1}\right) & \text{corr}\left(X_{2},X_{2}\right) & \cdots & \text{corr}\left(X_{2},X_{n}\right)\\ \vdots & \vdots & \ddots & \vdots \\ \text{corr}\left(X_{n},X_{1}\right) & \text{corr}\left(X_{n},X_{2}\right) & \cdots & \text{corr}\left(X_{n},X_{n}\right) \end{array}\right], \end{array}$$

where

(3) corr(Xi,Xj)=(Xi−X−)T(Xj−X−)(Xi−X−)T(Xi−X−)(Xj−X−)T(Xj−X−),

and $\overset{-}{X}$ is the sample mean of data matrix *X*.

Correlation is a mean-centered distance metric that is not common for kernel constructions. However, it is an important metric and problem specific. The usual correlation kernel is constructed based on the correlation matrix defined above. And the kernel value between *X*_*i*_ and *X*_*j*_ is

(4) $$\begin{array}{c} K\left(X_{i},X_{j}\right)=e^{-\gamma (1-\text{corr}(X_{i},X_{j}))}. \end{array}$$

This kind of kernel definition appropriately describes the similarity between two data instances. It is direct to see the symmetric property of the kernel matrix as well. To have a better understanding of the kernel matrix, we can describe it as follows:

(5) $$\begin{array}{c} K=\left[\begin{array}{cccc} 1 & k_{12} & \cdots & k_{1n}\\ k_{21} & 1 & \cdots & k_{2n}\\ \vdots & \vdots & \ddots & \vdots \\ k_{n1} & k_{n2} & \cdots & 1 \end{array}\right], \end{array}$$

where 0 < *k*_*ij*_ ≤ 1, *i*, *j* = 1,2,…, *n*. The following proposition presents relationship with corr(*X*).

**Proposition 1**

The usual correlation kernel is positive semidefinite if corr(*X*) is positive semidefinite.

**Proof**

The correlation kernel is symmetric and we have *k*_*ij*_ = *k*_*ji*_, *i*, *j* = 1,2,…, *n*. If we denote *k*_12_ = *a*, *k*_13_ = *b*, *k*_23_ = *c* then we have the following description of the kernel matrix:

(6) $$\begin{array}{c} K=e^{\left(-\gamma \right)}*e^{\left(\gamma *\text{corr}(X)\right)} \end{array}$$

Because *γ* > 0, we have

(7) $$\begin{array}{c} e^{\left(-\gamma \right)}>0. \end{array}$$

What's more,

(8) $$\begin{array}{c} e^{\left(\gamma *\text{corr}(X)\right)} \end{array}$$

has the same definite property with

(9) $$\begin{array}{c} e^{\text{corr}(X)}. \end{array}$$

Using kernel trick in machine learning area, we can see that if corr(*X*) is positive semidefinite, then usual correlation kernel is also positive semidefinite.

### 2.2. A Parsimonious Correlation Kernel

To deal with the positive semidefinite requirement of a kernel matrix, in this subsection, we propose a parsimonious kernel which is simply the correlation matrix *K*_*P*_ = corr(*X*). The proposition below shows that the proposed kernel is positive semidefinite.

**Proposition 2**

The matrix corr(*X*) is a positive semidefinite matrix.

**Proof**

From (3), we know that the *ij*th entry of corr(*X*) is given by

(10) $$\begin{array}{c} K_{P}\left(i,j\right)=\frac{{\left(X_{i}-\overset{-}{X}\right)}^{T}\left(X_{j}-\overset{-}{X}\right)}{\sqrt{{\left(X_{i}-\overset{-}{X}\right)}^{T}\left(X_{i}-\overset{-}{X}\right)}\sqrt{{\left(X_{j}-\overset{-}{X}\right)}^{T}\left(X_{j}-\overset{-}{X}\right)}}. \end{array}$$

Alternatively, we may write

(11) $$\begin{array}{c} K_{P}\left(i,j\right)=\langle \frac{\left(X_{i}-\overset{-}{X}\right)}{\sqrt{{\left(X_{i}-\overset{-}{X}\right)}^{T}\left(X_{i}-\overset{-}{X}\right)}},\frac{\left(X_{j}-\overset{-}{X}\right)}{\sqrt{{\left(X_{j}-\overset{-}{X}\right)}^{T}\left(X_{j}-\overset{-}{X}\right)}}\rangle . \end{array}$$

If we denote

(12) $$\begin{array}{c} T=\left[\frac{\left(X_{1}-\overset{-}{X}\right)}{\sqrt{{\left(X_{1}-\overset{-}{X}\right)}^{T}\left(X_{1}-\overset{-}{X}\right)}},\ldots ,\frac{\left(X_{n}-\overset{-}{X}\right)}{\sqrt{{\left(X_{n}-\overset{-}{X}\right)}^{T}\left(X_{n}-\overset{-}{X}\right)}}\right] \end{array}$$

from the separability of the kernel matrix, we can rewrite *K*_*P*_ = *T* ^*T*^ · *T*. Then for any

(13) $$\begin{array}{c} Y={\left[y_{1},y_{2},\ldots ,y_{n}\right]}^{T}\in R^{n}, \end{array}$$

we have

(14) $$\begin{array}{c} Y^{T}K_{P}Y={\left(TY\right)}^{T}\left(TY\right). \end{array}$$

If we further assume

(15) $$\begin{array}{c} Z=TY={\left[z_{1},z_{2},\ldots ,z_{n}\right]}^{T}, \end{array}$$

then

(16) $$\begin{array}{c} Y^{T}K_{P}Y=Z^{T}Z=\sum_{i=1}^{n}z_{i}^{2}\geq 0. \end{array}$$

This demonstrates that *K*_*P*_ = corr(*X*) itself is a parsimonious kernel matrix satisfying positive semidefinite property automatically.

Therefore, *K*_*P*_ can be employed as a kernel matrix for training classifiers in machine learning framework. This further proves the positive semidefiniteness of usual correaltion matrix.

### 2.3. Denoised Correlation-Based Kernel

From the successful experience of the usual correlation kernel in Raman Spectra classification, we construct a novel kernel utilizing the advantage of the usual correlation kernel. The denoised correlation-based kernel construction involves two steps. First, we formulate a kernel matrix sharing similar property of the usual correlation kernel. Second, *denoising* techniques are applied in order to construct a positive semidefinite kernel matrix. The above ideas can be summarized in the following two steps.

***Step *1* (a new kernel)***

Here we propose a new kernel having equivalent property with the usual correlation kernel. It is defined as follows:

(17) $$\begin{array}{c} K_{\text{CB}}=1-e^{-\text{corr}(X)}. \end{array}$$

Since

(18) $$\begin{array}{c} K_{\text{CB}}=1-e^{-\text{corr}(X)}, \end{array}$$

we can write it in another way as follows:

(19) $$\begin{array}{c} K_{\text{CB}}=\left(1-e^{-1}\right)K1_{\text{CB}}, \end{array}$$

where

(20) $$\begin{array}{c} K1_{\text{CB}}=\left[\begin{array}{cccc} 1 & \frac{1-e^{-\text{corr}(X_{1},X_{2})}}{1-e^{-1}} & \cdots & \frac{1-e^{-\text{corr}(X_{1},X_{n})}}{1-e^{-1}}\\ \frac{1-e^{-\text{corr}(X_{2},X_{1})}}{1-e^{-1}} & 1 & \cdots & \frac{1-e^{-\text{corr}(X_{2},X_{n})}}{1-e^{-1}}\\ \vdots & \vdots & \ddots & \vdots \\ \frac{1-e^{-\text{corr}(X_{n},X_{1})}}{1-e^{-1}} & \frac{1-e^{-\text{corr}(X_{n},X_{2})}}{1-e^{-1}} & \cdots & 1 \end{array}\right], \end{array}$$

has similar expression with the usual correlation kernel.

***Step *2* (the denoising strategy)***

In order to avoid the problem of nonpositive semidefiniteness of the kernel matrix, we incorporate *denoising* strategy in the kernel construction. Because *K*_CB_ = *V* ^*T*^ *PV*, where *V* ^*T*^ is the matrix composed of all the eigenvectors of the matrix *K*_CB_ and *P* is a diagonal matrix where the diagonal entries are the eigenvalues of the matrix *K*_CB_ then we denote it by

(21) $$\begin{array}{c} P=\left[\begin{array}{cccc} p_{1} & 0 & \cdots & 0\\ 0 & p_{2} & \cdots & 0\\ \vdots & \vdots & \ddots & \vdots \\ 0 & 0 & \cdots & p_{n} \end{array}\right]. \end{array}$$

The *denoising* strategy is to transform the diagonal matrix *P* to another diagonal matrix $\tilde{P}$,

(22) $$\begin{array}{c} \tilde{P}=\left[\begin{array}{cccc} {\tilde{p}}_{1} & 0 & \cdots & 0\\ 0 & {\tilde{p}}_{2} & \cdots & 0\\ \vdots & \vdots & \ddots & \vdots \\ 0 & 0 & \cdots & {\tilde{p}}_{n} \end{array}\right], \end{array}$$

where

(23) $$\begin{array}{c} {\tilde{p}}_{i}=\{\begin{array}{cc} 0, & p_{i}<0;\\ p_{i}, & p_{i}\geq 0. \end{array},\;i=1,2,\ldots ,n. \end{array}$$

Finally, $K_{\text{DCB}}=V^{T}\tilde{P}V$ is a positive semidefinite kernel matrix.

### 2.4. Materials

We prepared three publicly available data sets from libsvm [15] related to three types of cancer.

The first data set is related to colon cancer. In the data set, there are 22 normal and 40 tumor colon tissues. Each tissue is characterized by intensities of 2,000 genes with highest minimal intensity through the samples [16]. The preprocessing process has been done through instance-wise normalization to standard normal distribution. Then feature-wise normalization was performed to the standard normal distribution as well. In total there are 62 data instance with 2000 features. There are 40 positive data which means 40 exhibiting colon cancer, while 22 are normal.

The second data set is related to breast cancer. Similar to the first data set, the same preprocessing technique applied to the data normalization. Initially, there are 49 tumor samples. They are derived from the Duke Breast Cancer SPORE tissue resource. And they were divided into two groups: estrogen receptor positive and estrogen receptor-negative, via immunohistochemistry [17]. However, the classification results using immunohistochemistry and protein immunoblotting assay conflicted, 5 of them are then removed. Therefore, there are 44 data instances in total, 21 are negative and 23 are positive. The number of genes used to describe the tumor sample is 7129.

The third data set is related to leukemia cancer. Preprocessing for the data set is exactly the same as the previous two data sets. The data set was composed of 38 bone marrow samples, 27 of them are acute myeloid leukemia, and the remaining 11 are acute lymphoblastic leukemia [18]. Expression levels of 7129 genes are used to measure each data.

## 3. Numerical Experiments

We compare our proposed methods with the following three state-of-the-art methods.

**(i) Decision Tree**

Decision tree learning is a method commonly used in data mining. It employs a decision tree as a predictive model which maps observations about an item to conclusions about the item's target value. In these tree structures, leaves represent classifications and branches represent conjunctions of features that lead to those classifications.

**(ii) K-Nearest Neighborhood (KNN)**

The *K*-nearest neighbor algorithm is the simplest method among all machine learning algorithms. An object is classified by a majority vote of its neighbors, with the object being assigned to the class most common amongst its *K*-nearest neighbors (*K* is a positive integer, typically small). If *K* = 1, then the object is simply assigned to the class of its nearest neighbor.

**(iii) Support Vector Machines (SVMs)**

A support vector machine constructs a hyperplane or set of hyperplanes in a high- or infinite-dimensional space, which can be used for classification. A good separation is achieved by the hyperplane that has the largest functional margin that is the distance to the nearest training data points of any class.

In this study, we employed the KNN method with *K* = 1,5, 10 and the decision tree algorithm for comparison with our proposed parsimonious correlation kernel and denoised correlation-based kernel with SVM. The aim is to demonstrate superiority of our proposed kernels to the usual correlation kernel.

Tables 1, 2, and 3 present the prediction accuracy comparison in different algorithms. Here we introduce some state-of-the-art models for the purpose of comparison, they are the decision tree method and the KNN Algorithm. We employ the 5-fold cross-validation setting in the study. To get a relatively stable result, 10 times 5-fold cross-validation was performed and the accuracy was measured as the averaged accuracy over the 10 runs. The best performance is marked in bold size in the tables.

For colon cancer data set, decision tree exhibits inferior performance compared to KNN algorithm. However, both decision tree and the KNN algorithm cannot do better than the usual correlation kernel when *γ* = 1. For different values of *γ*, the performance of the usual correlation kernel differs widely. The best performance is achieved when *γ* = 1 is adopted. But when *γ* = 0.1 or 10, only around 0.65 accuracy was obtained.

For breast cancer data, decision tree performed better than KNN algorithm. The accuracy for decision tree is 0.7653, but for the KNN algorithm, the best result obtained is 0.7447 when *K* = 1 which is significantly less than 0.7653. But still they cannot catch up with the usual correlation kernel when *γ* = 1 that is 0.7914. Similar to colon cancer data set, when *γ* = 0.1 and 10, the usual correlation kernel demonstrated poorly, the accuracies are only 0.6642 and 0.4114, respectively. As a conclusion, in general the parsimonious correlation kernel and the denoised correlation-based kernel are the best two.

Finally for leukemia data set, the accuracies of the decision tree method and the KNN algorithm are higher than usual correlation kernel. They are all over 0.8000 while the best performance of the usual correlation kernel is 0.7868 when *γ* = 1, less than 0.8. However, both parsimonious correlation kernel and denoised correlation-based kernel can achieve over 0.9000 accuracy.

**Figure 1:**
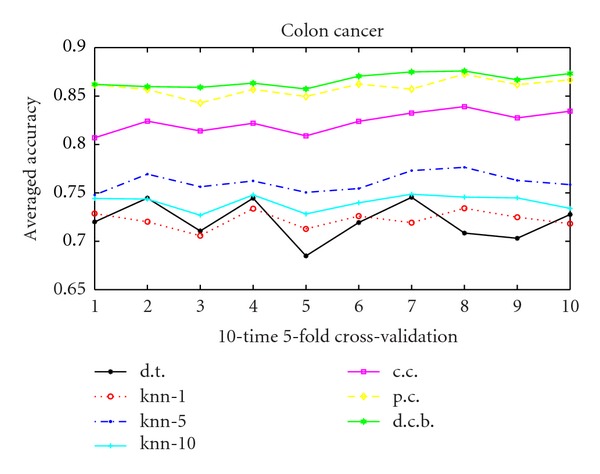
10 runs 10-time 5-fold cross-validation for colon cancer data. Solid line with black “∗” with legend d.t. is the decision tree method, dashed line with red “*⚪*” marked “knn-1^”^ means *K*-nearest neighborhood when *K* = 1, dash-dot line with blue point marked “knn-5” means *K*-nearest neighborhood when *K* = 5, solid line with “+” in cyan labeling “knn-10” is the *K*-nearest neighborhood when *K* = 10, solid line with magenta “□” is the usual correlation kernel, diamond yellow line represents the parsimonious correlation kernel, and hexagram green line is the denoised correlation-based kernel.

**Figure 2:**
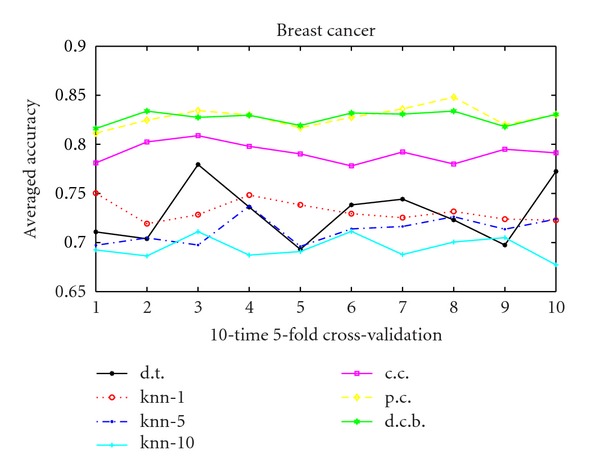
10 runs 10-time 5-fold cross-validation for breast cancer data. Solid line with black “∗” with legend d.t. is the decision tree method, dashed line with red “*⚪*” marked “knn-1” means *K*-nearest neighborhood when *K* = 1, dash-dot line with blue point marked “knn-5” means *K*-nearest neighborhood when *K* = 5, solid line with “+” in cyan labeling “knn-10” is the *K*-nearest neighborhood when *K* = 10, solid line with magenta “□” is the usual correlation kernel, diamond yellow line represents the parsimonious correlation kernel, and hexagram green line is the denoised correlation-based kernel.

**Figure 3:**
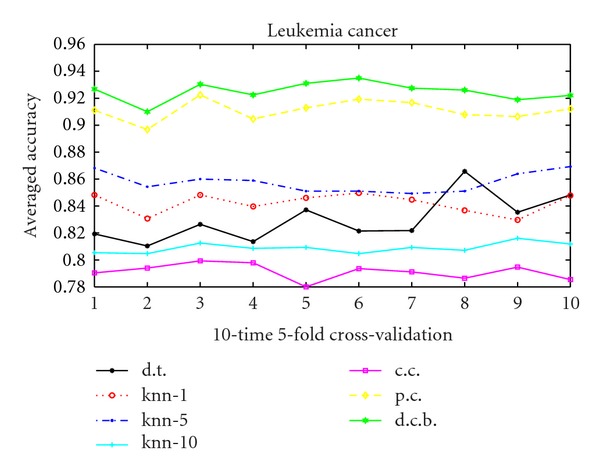
10 runs 10-time 5-fold cross-validation for leukemia cancer data. Solid line with black “∗” with legend d.t. is the decision tree method, dashed line with red “*⚪*” marked “knn-1” means *K*-nearest neighborhood when *K* = 1, dash-dot line with blue point marked “knn-5” means *K*-nearest neighborhood when *K* = 5, solid line with “+” in cyan labeling “knn-10” is the *K*-nearest neighborhood when *K* = 10, solid line with magenta “□” is the usual correlation kernel, diamond yellow line represents the parsimonious correlation kernel, and hexagram green line is the denoised correlation-based kernel.

As we can conclude that for the usual correlation kernel, *γ* = 1 ensures the best performance. Hence we choose *γ* = 1 in the following studies. Figures 1, 2, and 3 show the performance of 10 runs of 10-time 5-fold cross-validation for the 3 data sets. Value *i* in *X*-label means the *i*th run. And *Y*-label means the averaged accuracy of each 10-time 5-fold cross-validation. We compare the decision tree method, the KNN algorithm, the usual correlation kernel and the 2 proposed positive semidefinite kernels: Parsimonious correlation kernel and denoised correlation-based kernel. The figures clearly demonstrate the superiority of the our 2 proposed kernels (as presented in starred green and diamond yellow in the figures) over all the other algorithms compared.

Table 4 presents the dominant eigenvalues in PC kernel and DCB kernel. We observe that the dominant eigenvalues for PC kernel and DCB kernel are very close to each other, with a gap of only 0.0168. This explain why the two algorithms exhibit similar performance. And for the colon cancer data set, the difference in dominant eigenvalues is 0.1421. While for the leukemia data set, the difference is the largest: 0.3048. One can see that the performance difference is also the largest, the superiority of DCB kernel over PC kernel is the clearest. The difference in the dominant eigenvalues is consistent with the difference in performance in classification. The larger the difference in eigenvalues, the larger the difference in classification performance will be.

## 4. Discussions

From the tables, one can see the consistent superiority of the denoised correlation-based kernel for classification. All of them can achieve the best for the 3 tested data sets. And the positive semidefinite Parsimonious kernel is the second best among all the algorithms compared. Moreover, we observe no dominant superiority for decision tree or KNN algorithm over the other.

From the perspective of the usual correlation kernel, in the colon cancer data, it is better compared to decision tree and KNN algorithm, the average accuracy is located around 0.8000 while decision tree and KNN algorithm cannot exceed 0.7500 in general. In the breast cancer data, similar conclusions can be drawn for the usual correlation kernel. Second to our proposed PC kernel and DCB kernel, it ranks 3 in all the investigated methods. But in the leukemia data set, UC kernel is the lowest in accuracy. It cannot compete with all the other methods presented. This concludes that there is also no dominant advantage of the UC kernel over the decision tree method and the KNN algorithm.

If we focus on the comparison of the 2 proposed positive semidefinite kernels: PC kernel and DCB kernel, we can also reach some conclusions. For breast cancer data, the two show comparable performance. But for colon cancer data and leukemia data, DCB kernel demonstrates its superiority. The superiority is much clearer in leukemia data set. The reasons explaining the difference can be possibly given by the dominant eigenvalue theory. In finance, the largest eigenvalue gives a rough idea on the largest possible risk of the investment in the market [19]. The dominant eigenvalue is the one provides the most valuable information about the dynamics from which the matrix came from [20].

## 5. Conclusions

In this study, two positive semidefinite kernels which we call parsimonious correlation kernel and denoised correlation-based kernel have been proposed in discriminating different tumor tissues, offering diagnostic suggestions. We have provided theoretical illustrations on the positive semidefinite property of the usual correlation kernel. Taking into consideration of the positive semidefiniteness of correlation matrix, we have proposed 2 positive semidefinite kernels. The robustness of the 2 proposed kernels in conjunction with support vector machines is demonstrated through 3 publicly available data sets related to cancer in tumor discrimination. Comparisons with the state-of-the-art methods like the decision tree method and the KNN algorithm are made. Investigation on the performance analysis for the 2 proposed positive semidefinite kernels is conducted with eigenvalue theory support. The proposed kernels highlight the importance of positive semidefiniteness in kernel construction. As novel kernels using distance metric for kernel construction that are not common in machine learning framework, the proposed kernels are hoping to be applied in a wider range of areas.

## Figures and Tables

**Table 1:** Prediction accuracy comparison among different algorithms for colon cancer.

DT	UC			PC	DCB	KNN		
	*γ* = 0.1	*γ* = 1	*γ* = 10			*K* = 1	*K* = 5	*K* = 10
0.6945	0.6623	0.8059	0.6459	0.8490	0.8599	0.7244	0.7519	0.7404

Here DT means decision tree algorithm, UC means the usual correlation kernel, PC stands for the parsimonious correlation we proposed, DCB is the novel kernel proposed by us representing denoised correlation-based kernel, and KNN is the *K*-nearest neighborhood algorithm.

**Table 2:** Prediction accuracy comparison among different algorithms for breast cancer.

DT	UC			PC	DCB	KNN		
	*γ* = 0.1	*γ* = 1	*γ* = 10			*K* = 1	*K* = 5	*K* = 10
0.7653	0.6642	0.7914	0.4114	0.8075	0.8278	0.7447	0.7147	0.6872

Here DT means decision tree algorithm, UC means the usual correlation kernel, PC stands for the parsimonious correlation we proposed, DCB is the novel kernel proposed by us representing denoised correlation-based kernel, and KNN is the *K*-nearest neighborhood algorithm.

**Table 3:** Prediction accuracy comparison among different algorithms for Leukemia cancer.

DT	UC			PC	DCB	KNN		
	*γ* = 0.1	*γ* = 1	*γ* = 10			*K* = 1	*K* = 5	*K* = 10
0.8189	0.7111	0.7868	0.7111	0.9150	0.9257	0.8443	0.8557	0.8082

Here DT means decision tree algorithm, UC means the usual correlation kernel, PC stands for the parsimonious correlation we proposed, DCB is the novel kernel proposed by us representing denoised correlation-based kernel, and KNN is the *K*-nearest neighborhood algorithm.

**Table 4:** Dominant eigenvalues for parsimonious correlation Kernel and Denoised Correlation Based Kernel.

	Colon cancer	Breast cancer	Leukemia cancer
P.C.	8.9108	8.7721	5.2066
D.C.B.	9.0529	8.7889	5.5114

Here PC stands for the parsimonious correlation we proposed, DCB is the novel kernel proposed by us representing denoised correlation-based kernel.
